# Olmesartan Decreased Levels of IL-1β and TNF-α, Down-Regulated MMP-2, MMP-9, COX-2, RANK/RANKL and Up-Regulated SOCs-1 in an Intestinal Mucositis Model

**DOI:** 10.1371/journal.pone.0114923

**Published:** 2014-12-22

**Authors:** Raimundo Fernandes de Araújo Júnior, Maria Patrícia Oliveira da Silva Reinaldo, Gerly Anne de Castro Brito, Pedro de França Cavalcanti, Marco Aurélio de Moura Freire, Caroline Addison Xavier de Medeiros, Aurigena Antunes de Araújo

**Affiliations:** 1 Post graduation program Health Science/Department of Morphology, UFRN, Natal, RN, Brazil; 2 Post graduation program in Functional and Structural Biology/UFRN, Natal, RN, Brazil; 3 Post graduation program in Pharmacology/Department of Morphology/UFC, Fortaleza, CE, Brazil; 4 Department of Morphology/UFRN, Natal, RN, Brazil; 5 Edmond and Lily Safra International Institute of Neuroscience of Natal (ELS-IINN), Natal, RN, Brazil; 6 Department of Biophysics and Pharmacology, UFRN, Natal, RN, Brazil; 7 Post graduation program Public Health/Department of Biophysics and Pharmacology/UFRN, Natal, RN, Brazil; 8 Post graduation program in Pharmaceutical Science/UFRN, Natal, RN, Brazil; Innsbruck Medical University, Austria

## Abstract

Methotrexate (MTX) is a pro-oxidant compound that depletes dihydrofolate pools and is widely used in the treatment of leukaemia and other malignancies. The efficacy of methotrexate is often limited by mucositis and intestinal injury, which are major causes of morbidity in children and adults. The aim of this study was to evaluate the effect of olmesartan (OLM), an angiotensin II receptor antagonist, on an Intestinal Mucositis Model (IMM) induced by MTX in Wistar rats. IMM was induced via intraperitoneal (i.p.) administration of MTX (7 mg/kg) for three consecutive days. The animals were pre-treated with oral OLM at 0.5, 1 or 5 mg/kg or with vehicle 30 min prior to exposure to MTX. Small intestinal homogenates were assayed for levels of the IL-1β, IL-10 and TNF-α cytokines, malondialdehyde and myeloperoxidase activity. Additionally, immunohistochemical analyses of MMP-2, MMP-9, COX-2, RANK/RANKL and SOCS-1 and confocal microscopy analysis of SOCS-1 expression were performed. Treatment with MTX + OLM (5 mg/kg) resulted in a reduction of mucosal inflammatory infiltration, ulcerations, vasodilatation and haemorrhagic areas (p<0.05) as well as reduced concentrations of MPO (p<0.001) and the pro-inflammatory cytokines IL-1β (p<0.001) and TNF-a (p<0.01), and increase anti-inflammatory cytocine IL-10 (p<0.05). Additionally, the combined treatment reduced expression of MMP-2, MMP-9, COX-2, RANK and RANKL(p<0.05) and increased cytoplasmic expression of SOCS-1 (p<0.05). Our findings confirm the involvement of OLM in reducing the inflammatory response through increased immunosuppressive signalling in an IMM. We also suggest that the beneficial effect of olmesartan treatment is specifically exerted during the damage through blocking inflammatory cytocines.

## Introduction

Oral and gastrointestinal mucositises are common complications of chemotherapy, in particular with drugs affecting DNA synthesis (S-phase-specific agents such as fluorouracil, methotrexate, and cytarabine). Mucositis occurs in 40% of patients after standard dose chemotherapy, and in 100% of patients undergoing high dose chemotherapy and stem cell or bone marrow transplantation and contributes not only to the morbidity of treatment but also to its cost [1]. The pathogenesis of chemotherapy induced gastrointestinal mucositis includes five phases: initiation by chemotherapy, up-regulation and generation of messenger signals, signaling by pro-inflammatory cytokines and amplification of mucosal injury, ulceration of the mucosa and finally, healing. The initial stages of inflammation in mucositis include increased pro-inflammatory cytokine levels, which act as a homing marker for inflammatory immune cells in the submucosa [2].

Methotrexate (2,4-diamino-N10-methyl propylglutamic acid, MTX) is one of the most widely studied therapeutics agents available to treat many solid tumors, hematologic malignancies, and autoimmune diseases [3]. MTX acts as a cancer chemotherapeutic agent by inhibiting dihydrofolate reductase (DHFR) with high affinity, resulting in depletion of tetrahydrofolates that are required for the synthesis of of DNA and RNA [4]. However, in addition to cancer cells being affected by MTX, rapid proliferating cells such as bone marrow and gastrointestinal cells are also affected. One of the most important side effects of MTX is related to the gastrointestinal tract. [5], [6].

Mucositis is typically accompanied by oral and/or abdominal pain, ulceration, dysphagia, and diarrhea, which often result in communication impairment, reduction in fluid and food intake, and consequent dehydration and weight loss [7].

The use of bioactive/growth factors, hormones or interleukins to modify epithelial metabolism and reduce the susceptibility of the tract to mucositis [8]. Some of these treatments appear to have considerable potential and are at present under clinical evaluation. Presently available treatments do not prevent mucositis, but can limit its severity if used in combination.

Cancer patients may have systemic diseases, as hypertension arterial, that are treated in parallel to chemotherapy, and that can mitigate or aggravate the adverse effects of chemotherapy during treatment [9]. Our group has studied the angiotensin II receptor blocker (ARB). For example, the angiotensin II receptor blocker (ARB) has been implicated as an anti-inflammatory agent that suppresses tumor necrosis factor (TNF)-α-induced activation of nuclear factor (NF)-κB in vascular endothelial cells [10]. In experimental model, Telmisartan, angiotensin II receptor blocker (ARB), reduced markers of inflammation, proteases and changed proteins involved in bone remodeling [11]. Similar results were obtained in a study using another ARB, olmesartan [12].

The goal of this study was show anti-inflammatory activity of olmesartan in model experimental mucositis intestinal.

## Materials and Methods

### Chemicals

Methotrexate was purchased from LIBBs Farmacêutica Ltda, São Paulo, Brazil. Olmesartan medoximil (Benicar 20 mg, Daiichi Sankyo Brazil Farmacêutica Ltda, São Paulo, Brazil), O-Dianisine Sigma (São Paulo, Brazil), antibodies (Santa Cruz Biotechnology, INTERPRISE, Brazil): COX-2; MMP-2; MMP-9; RANK; RANKL; SOCS-1, Streptavidin-HRP-conjugated secondary antibody (Biocare Medical, Concord, CA, USA). TrekAvidin-HRP Label + Kit from Biocare Medical, Dako, USA. IL-1β, IL-10, TNF-α ELISA kit (R&D Systems, Minneapolis, MN, USA).

### Animals

Experiments were performed on male Wistar albino rats weighing between 250 and 300 g, purchased from Bioterio Department of Biophysical and Pharmacology. Animals were housed in a temperature and humidity controlled environment under a 12-h light/dark cycle (lights on at 6 AM). Food and water were available *ad libitum*. The animals used in the experiments originated from the Department of Biophysics and Pharmacology. The animals were housed individually in polypropylene cages measuring 41×34×16 cm Autoclaved. Various signals of the health of the animals were monitored, including: coat condition, response to stimuli, faeces and urine. Only those animals in perfect health were kept in the experiment. Standard diet (Basic Composition: Soybean meal, dextrin, rice husks, wheat bran, rice bran, meat meal, fish meal, sodium chloride, magnesium oxide, iron sulfate, copper sulfate, manganese monoxide, zinc oxide, calcium iodate, cobalt sulphate, sodium selenite, vitamin A, vitaminD3, vitamin E, vitaminK3, vitamin B1, vitaminB2, niacin, pantothenic acid, vitaminB6, folic acid, biotin, vitamin B12, choline chloride, lysine, methionine, propionic acid, *Agrobacteriumtumefaciens*, and *Bacillusthuringiensis*; Presence/Evialis do Brasil Nutrição Animal LTDA, São Paulo) and water source (bottled). The National Institutes of Health Guidelines for the Care and Use of Laboratory Animals were followed. All efforts were made to minimize the number of animals used and their suffering degree. The methods used in this investigation were approved by CEUA/UFRN (approval number: 016/2013).

### Induction of experimental intestinal mucositis

Eighty rats were randomly divided into eight groups (five animals per group, duplicate groups). The vehicle control group received normal saline orally by gastric gavage and i.p saline (0.9% NaCl) for 3 days The positive control group received saline orally by gastric gavage and i.p. MTX (7 mg/kg) for 3 days. Three groups received oral OLM at 0.5, 1 and 5 mg/kg, respectively, by gastric gavage and i.p. saline (0.9% NaCl) for 3 days. Three additional groups received oral OLM at 0.5, 1 and 5 mg/kg, respectively, by gastric gavage and i.p. MTX (7 mg/kg) for 3 days. Animals were euthanized on the fourth day with (80 mg/kg, i.p.) 2% thiopental (Cristália, São Paulo, Brazil).

Following euthanasia, a cardiac puncture was performed and blood samples were taken for leukogram, bacteraemia and biochemical analyses. The small intestines of the rats were frozen at −80°C for analyses of cytokine, myeloperoxidase (MPO) and malonyldialdehyde levels. Intestines were immersed in 10% buffered formalin for histopathological analysis.

### Myeloperoxidase (MPO) assay

The extent of neutrophil accumulation in the small intestine samples was measured by assaying MPO activity. Intestinal mucosa (duodenum, three samples per group; jejunum, four samples per group; ileum, three samples per group) were harvested as described above and stored at −70°C until required for assay. After homogenisation and centrifugation (2000×*g* for 20 min), MPO activity was determined by a previously described colorimetric method [13]. Results are reported as units of MPO per gram of tissue.

### Malonyldialdehyde (MDA) assay

Malonyldialdehyde (MDA) is an end product of lipid peroxidation. To quantify the increase in free radicals in intestinal tissue samples, MDA content was measured via the assay described by Esterbauer and Cheeseman [14]. Samples (duodenum, three samples per group; jejunum, four samples per group; ileum, three samples per group) were suspended in buffer Tris HCl 1∶5 (w/v) and minced with scissors for 15 sec on an ice-cold plate. The resulting suspension was homogenised for 2 min with an automatic Potter homogenizer and centrifuged at 2500×g at 4°C for 10 min. The supernatants were assayed to determine MDA content. The results are expressed as nanomoles of MDA per gram of tissue.

### IL-1β, IL-10, and TNF-α assay

The intestinal tissue samples (duodenum, three samples per group; jejunum, four samples per group; ileum, three samples per group) were stored at −70°C until use. The tissue was homogenised and processed as described by Safieh-Garabedian, et al (1995) [15]. Levels of IL-1β (detection range: 62.5–4000 pg/mL; sensitivity or lower limit of detection [LLD]: 12.5 ng/mL of recombinant mouse IL-1β), IL-10 (detection range: 62.5–4000 pg/mL; sensitivity or LLD: 12.5 ng/mL of recombinant mouse IL-10) and TNF-α (detection range: 62.5–4000 pg/mL; sensitivity or LLD: 50 ng/mL of recombinant mouse TNF-α) in the intestinal samples were determined with a commercial ELISA kit (R&D Systems, Minneapolis, MN, USA), as described previously [16]. All samples were within the wavelength used in UV-VIS spectrophotometry (absorbance measured at 490 nm).

### Leukogram

Animals were anesthetized on day 4 with 2% thiopental (80 mg/kg, i.p.), and a blood sample was collected via heart puncture. Then, 20 µl of blood were added to 380 µl of Turk's solution. The total and differential counts of leukocytes were obtained by standard manual procedures using light microscopy [17]. The results are expressed as the number of cells per milliliter (ml).

### Bacteremia

On day 4, blood samples were collected via heart puncture under sterile conditions. Ten microlitres of blood were diluted tenfold in brain–heart infusion (BHI) medium. Bacterial growth was analysed after 24–48 h at 37°C by visual analysis of the turbidity of the culture medium. A turbid medium indicates bacteraemia (+), and a non-turbid medium suggests absence of bacteria in the blood. Organisms that grew on BHI medium were streaked for isolation on 5% sheep blood agar plates, MacConkey agar plates and mannitol-salt-agar plates. Organisms that grew were identified by standard methods, including Gram stain morphology, DNAse, catalase, coagulase, motility, sugar metabolism analyses.

### Histopathological analysis

The small intestines were excised quickly and washed with cold isotonic saline. Each segment was weighed and cut longitudinally. Three sections of small intestine (five animals per group) were analyzed. The specimens were fixed in 10% neutral buffered formalin, dehydrated and embedded in paraffin. Sections of 5 µm thickness were obtained for haematoxylin–eosin staining (H&E) and examined by light microscopy (40x, Olympus BX50, Morphology Department/UFRN). The parameters of inflammatory cell infiltration, vasodilatation, and the presence of haemorrhagic areas, oedema, ulcerations and abscesses were determined in a single-blind fashion and graded as follows: Score 1 - normal epithelium and connective tissue without vasodilatation; absence of or discreet cellular infiltration; and absence of haemorrhagic areas, ulcerations or abscesses; Score 2 - discreet vasodilatation and areas of re-epithelisation; discreet inflammatory infiltration with mononuclear prevalence; and absence of haemorrhagic areas, oedema, ulcerations or abscesses; Score 3 - moderate vasodilatation, areas of hydropic epithelial degeneration; inflammatory infiltration with neutrophil prevalence; and presence of haemorrhagic areas, oedema and eventual ulcerations, and absence of abscesses; and Score 4 - severe vasodilatation; inflammatory infiltration with neutrophil prevalence; and presence of haemorrhagic areas, oedema, ulcerations, and abscesses [18].

### Immunohistochemical analysis of MMP-2, MMP-9, COX-2, RANK, RANKL and SOCS-1

Thin sections of jejunum (4 µm) were obtained from each group (negative control, MTX and MTX-OLM 5 mg/kg) with a microtome and transferred to gelatine-coated slides. Each tissue section was then deparaffinised and rehydrated. The intestinal tissue slices were washed with 0.3% Triton X-100 in phosphate buffer (PB) and quenched with endogenous peroxidase (3% hydrogen peroxide). Tissue sections were incubated overnight at 4°C with 1∶400 dilutions of primary antibodies (Santa Cruz Biotechnology, INTERPRISE, Brazil) against COX-2, MMP-2, MMP-9, SOCS-1, RANK and RANKL. Dilution tests (3 dilutions) were performed with all antibodies to identify the 1∶400 dilution as appropriate. Slices were washed with phosphate buffer and incubated with a streptavidin/HRP-conjugated secondary antibody (Biocare Medical, Concord, CA, USA) for 30 minutes. Immunoreactivity to the various proteins was visualised with a colorimetric-based detection kit following the protocol provided by the manufacturer (TrekAvidin-HRP Label + Kit from Biocare Medical, Dako, USA). Sections were counter-stained with hematoxylin. Known positive controls and negative controls were included in each set of samples. Planimetry microscopy (Olympus BX50, Morphology Department/UFRN) with a high-power objective (40×) was utilised to score the intensity of cell immunostaining: 1 =  absence of positive cells; 2 =  small number of positive cells or isolated cells; 3 =  moderate number of positive cells; and 4 =  large number of positive cells. Labelling intensity was evaluated by two previously trained examiners in a double-blind fashion. Three tissue sections per animal (five animals per group) were evaluated.

### Analysis for confocal immunofluorescence

Three sections per animal (five animals per group) were immunostained with fluorescently labelled antibodies to reveal the SOCS-1 reactivity pattern. Briefly, sections were removed from the freezer, brought to room temperature for 10 min and washed for 10 minutes in PB. They were pre-treated for 25 min in 60°C 0.2 M boric acid (pH 9.0). Sections were cooled for about 20 min and washed in 0.1 M PB-Tween for 10 minutes (2×5 minutes), followed by incubation in 10% normal goat serum (Vector Laboratories, USA) for 30 minutes. The sections were incubated overnight with rabbit SOCS-1 primary antibody (1∶400 in 1% normal goat serum; Santa Cruz Biotechnology, USA) at 18°C, washed in 0.1 M PB-T for 10 minutes (2×5 minutes) and incubated with Alexa Fluor 488-conjugated goat anti-rabbit secondary antibody (1∶700 in 0.1 M PB, Invitrogen, Grand Island, NY, USA). Finally, the sections were mounted using Vectashield mounting medium for fluorescence (DAPI/Antifade solution, Vector Laboratories, USA). Fluorescent images were obtained on a Carl Zeiss Laser Scanning Microscope (LSM 710, 20x and 63x objectives, Carl Zeiss, Jena, Germany, Edmond and Lily Safra International Institute of Neuroscience of Natal, Brazil).

Known positive and negative controls were included in each batch of samples. Tissue reactivity in all groups (negative control, MTX and MTX-OLM 5 mg/kg) was assessed by computerised densitometry analysis using digital images captured with the confocal microscope. Average densitometric values were obtained using the *ImageJ* software (http://rsb.info.nih.gov/ij/). Measurements were obtained from a 0.02 mm^2^ square window positioned across the regions of interest (mucosa, three samples per animal). To minimize the effects of within-group variability, we utilised a normalized scale based on the underlying muscle layer (averaged over measurements of three different sites using the same window). For every animal, the average optical density (OD) for the regions of interest (mucosa) was designated R. The OD of the underlying muscle layer was designated L. To compare SOX reactivity between all three groups, a contrast index was calculated according to the equation: C =  (R - L)/(R+L) (modified from Freire *et al*) [19].

### Statistical analysis

Data are presented as means with standard errors of the mean (SEMs) or as medians, when appropriate. Analysis of variance (ANOVA) followed by Bonferroni's test was used to calculate the means. The Kruskal-Wallis test followed by Dunn's test was used to compare medians (GraphPad Prism 5.0 Software, La Jolla, CA, USA). A p-value of <0.05 indicated a statistically significant difference.

## Results

### Myeloperoxidase and malonyldialdehyde activity

**Figure 1:**
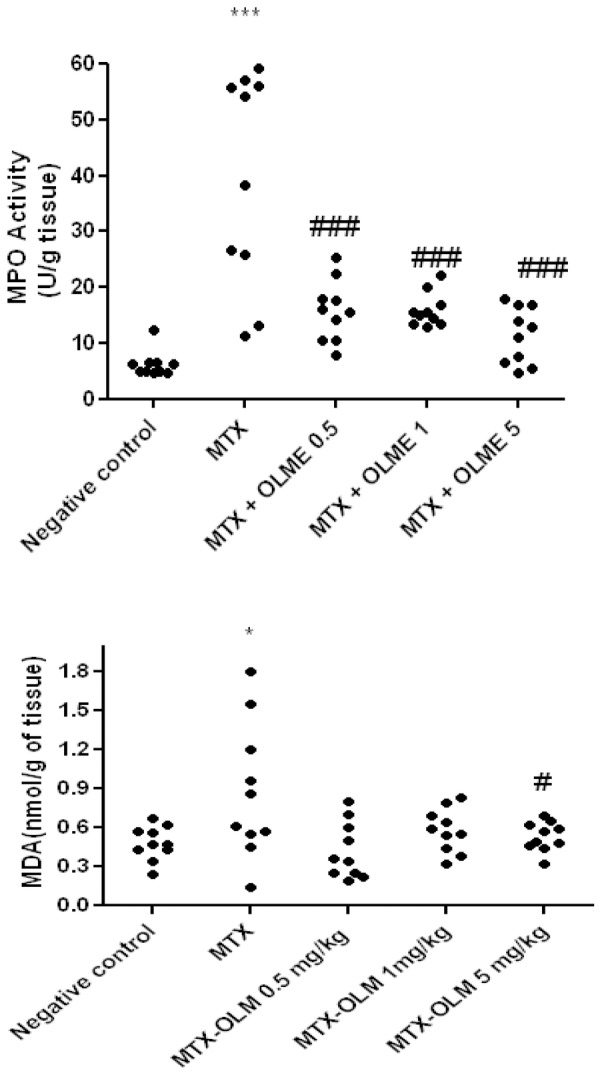
OLM attenuates MTX effects on MPO and MDA. The MTX alone group had significantly greater MPO and MDA than the Negative control (***p<.001 and *p<0.5, respectively). All three doses of OLM (0.5 mg/kg, 1 mg/kg, 5 mg/kg) countered the MTX effect on MPO significantly (###*p*<.001 *vs*. MTX). Although all doses of OLM appeared to reduce MDA subjectively, only the highest dose (5 mg/kg) produced a significant difference versus the MTX alone group (#*p*<.05). Segments of duodenum, jejunum and ileum/Duplicate Experiments.

MPO activity was measured in the small intestine tissue. The MTX alone group had significantly greater MPO and MDA than the Negative control (*p*<.001 and p<0.5, respectively). Levels of MPO in all olmesartan treatment groups with MTX were significantly decreased (p<0.001) compared to the MTX alone group . Levels of MDA in Negative control and MTX-OLM 5 mg/kg were significantly decreased (p<0.05) (Fig. 1).

### Effect of treatment on levels of IL-1β, IL-10 and TNF-α inflammatory activity

**Figure 2:**
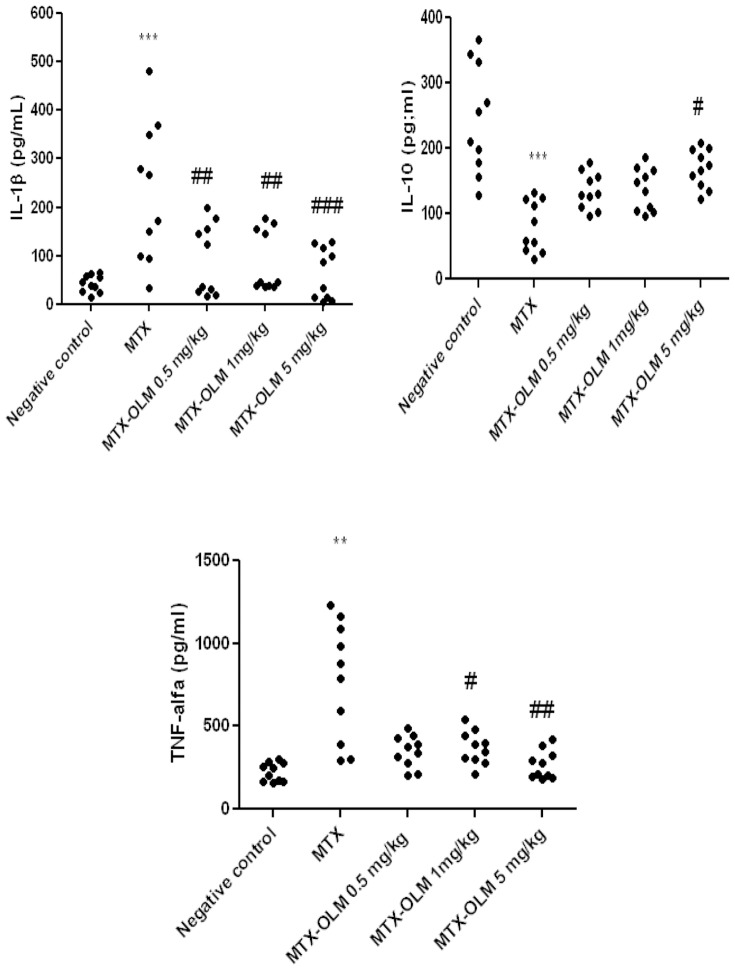
OLM opposes MTX influence on cytokines. MTX elevated levels of IL-1β (***p<0.001) and TNF-α (**p<0.01), while reducing IL-10 levels(***p<0.001) than the Negative control. OLM had dose-dependent MTX-countering effects on IL-1β (0.5 mg/kg, 1 mg/kg ##p<0.01 and 5 mg/kg ### p<0.001) and TNF-α (1 mg/kg #p<0.05 and 5 mg/kg ## p<0.01), but only OLM 5 mg/kg a significant MTX-countering effect on IL-10 levels (#*p*<0.05). Segments of duodenum, jejunum and ileum/Duplicate Experiments.

MTX elevated levels of IL-1β (p<0.001) and TNF-α (p<0.01), while reducing IL-10 levels (p<0.001) than the Negative control. The IL-1β and TNF-α levels were decreased in the MTX-OLM 5 mg/kg (p<0.001 and p<0.01), MTX-OLM 1 mg/kg (p<0.001 and p<0.05, respectively). Levels of IL-1β were significantly decreased in the MTX-OLM 0.5 mg/kg (p<0.001) group, compared to the MTX group. Levels of the anti-inflammatory cytokine IL-10 differ significantly between the MTX-OLM 5 mg/kg group and the MTX group, *p*<.05 (Fig. 2).

### Effects of OLM on leukocytosis and bacteraemia

**Figure 3:**
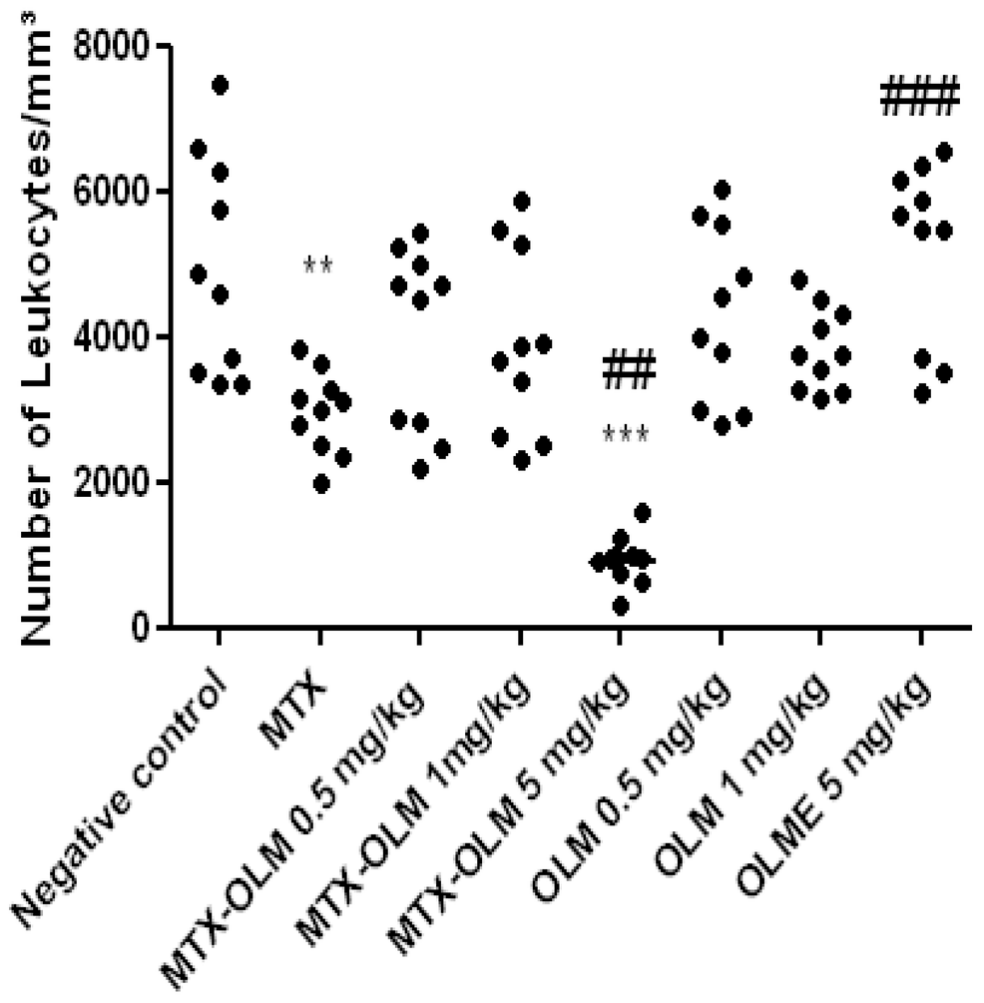
MTX and OLM effects on leukocyte density. MTX alone and MTX-OLM 5 mg/kg reduced leukocyte density (**p<.001 and *** p<0.001, respectively *vs*. Negative control), indeed, the and MTX-OLM 5 mg/kg resulted in an even stronger reduction effect than MTX alone (##*p*<.01 *vs*. MTX). OLM 5 mg/kg increased leukocyte density significantly (###*p*<.001 *vs*. MTX).

We observed significant leukopenia in animals treated with MTX (p<0.001) and MTX-OLM 5 mg/kg (p<0.001, Fig. 3) with number of leukocytes less than 2000/mm^3^ cells, indeed, the MTX-OLM 5 mg/kg resulted in an even stronger reduction effect than MTX alone (##*p*<.01 *vs*. MTX). OLM 5 mg/kg increased leukocyte density significantly (###*p*<.001 *vs*. MTX), Fig. 3. The blood culture results were negative for all groups, indicating the absence of bacteraemia (data not shown).

### Histological analysis

**Figure 4:**
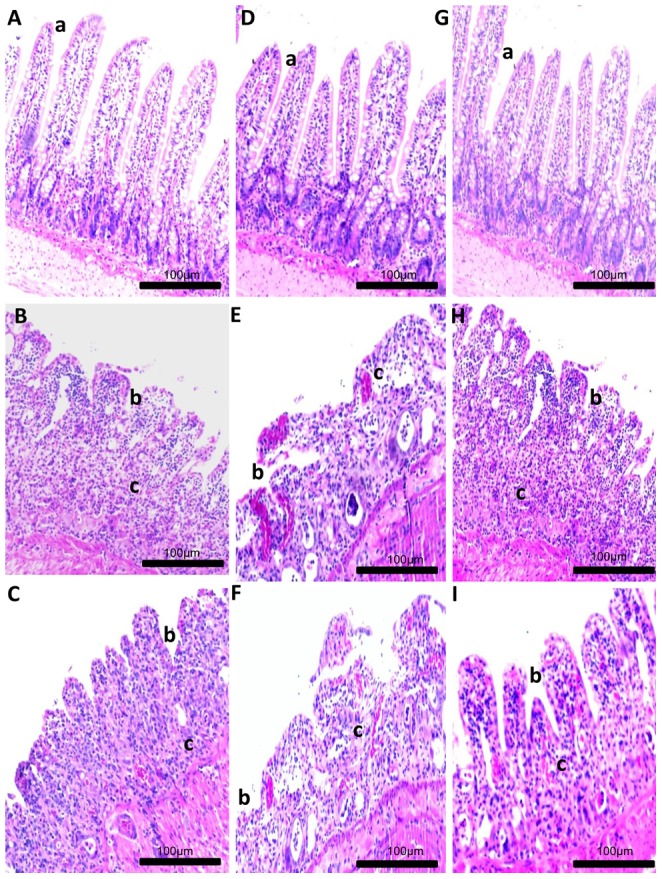
Histological examination of jejunum specimens. For each group, 5 animals were used, and three H&E sections from each animal were analyzed. Representative samples from each OLM treatment group are shown with graphs summarizing the group's mean histopathological score . Images and data from the low, middle, and high OLM dose groups are shown in panels A–C; D–F; G–I respectively (micrograph key: **a**, villus height; **b**, reduction of villus; **c**, inflammatory infiltrate). Control group slides are shown in A, D, and G (upper left image in each group). MTX alone group slides are shown in panels B, E, and H(middle images). Note that the MTX alone rats' jejunum exhibited intestinal mucositis with loss of crypt architecture, severe villous epithelial atrophy, degeneration and shortening of the villus length, and polymorphonuclear leukocyte infiltration in the lamina propria. Damage in mucosa as intense inflammation and villous epithelial atrophy persisted in the MTX-OLM 0.5 mg/kg (C) and MTX-OLM 1 mg/kg (F) groups (# p>.05 *vs.* MTX). (I) Conversely, reduced inflammation, reepithelization with decreased vasodilatation, decreased cellular infiltration, and reduction of hemorrhagic areas, ulcerations, and abscesses were observed in the jejunum from animals treated with MTX-OLM 5 mg/kg for 3 d. Magnification 20×, scale bar  = 100 µm.

**Figure 5:**
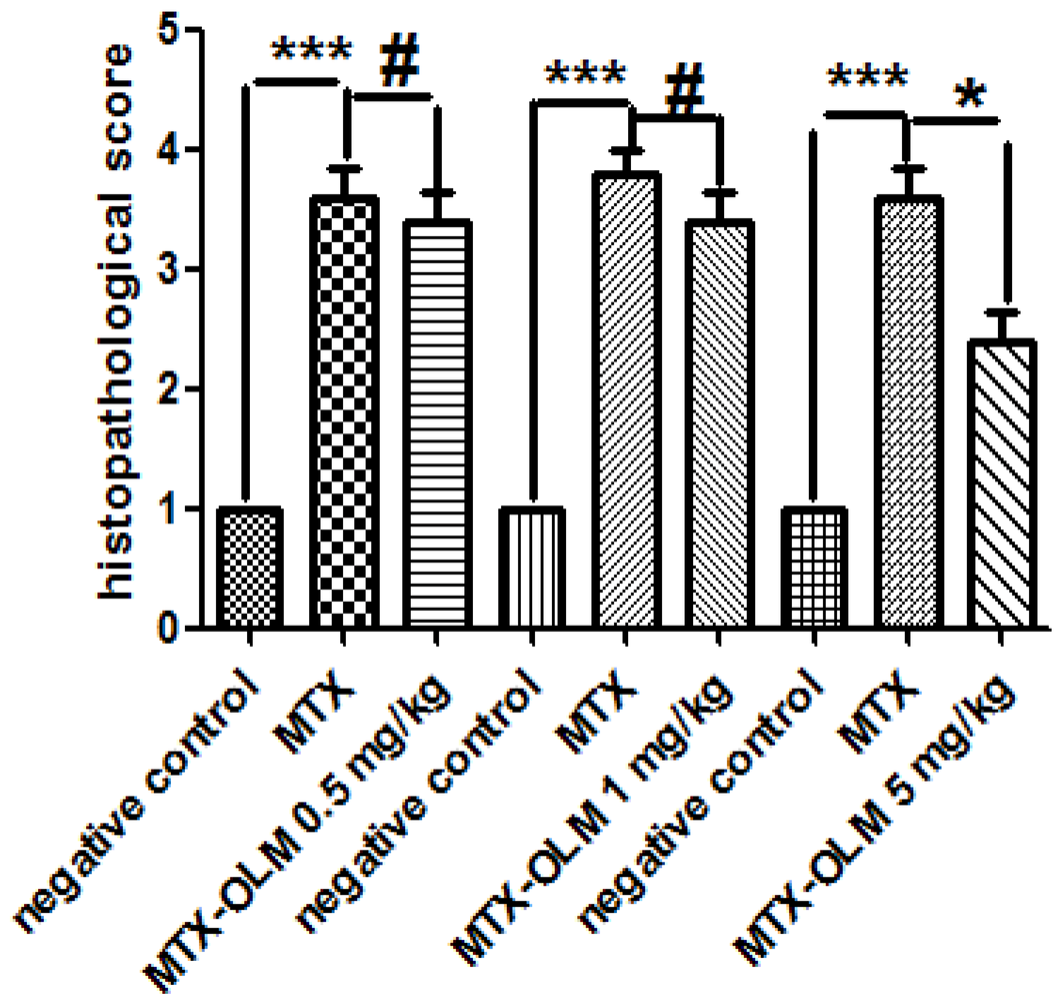
Effect of OLM treatment on intestinal damage in rats. For each group, 5 animals were used, and three H&E sections from each animal were analyzed. Representative samples from each OLM treatment group are shown with graphs summarizing the group's mean histopathological score. Values are expressed as means ± SEM (Compared to negative control ***p<0.001, compared to MTX *p<0.05; compared to MTX #*p*>0.05).

MTX-treated animals histologically exhibited significant loss of crypt architecture and signs of crypt remodelling, severe villous epithelial atrophy, degeneration and shortening of the villus, and polymorphonuclear leukocyte infiltration in the lamina propria. Histological damage was initially assessed in the jejunum with a semi-quantitative score. Intestinal damage was reduced in animals treated with MTX- OLM 5 mg/kg (p<0.05, Figs. 4 I and 5) compared with animals that received mg/kg MTX-OLM 0.5 or 1 mg/kg (p>0.05, Fig. 4 C and F, respectively and Fig. 5). These results were histopathologically obvious in the MTX-OLM 5 mg/kg group, which exhibited reduced tissue damage, preserved areas of the villus, and reductions of cellular infiltration and areas of haemorrhage or ulceration.

### Imunohistochemical analysis

**Figure 6:**
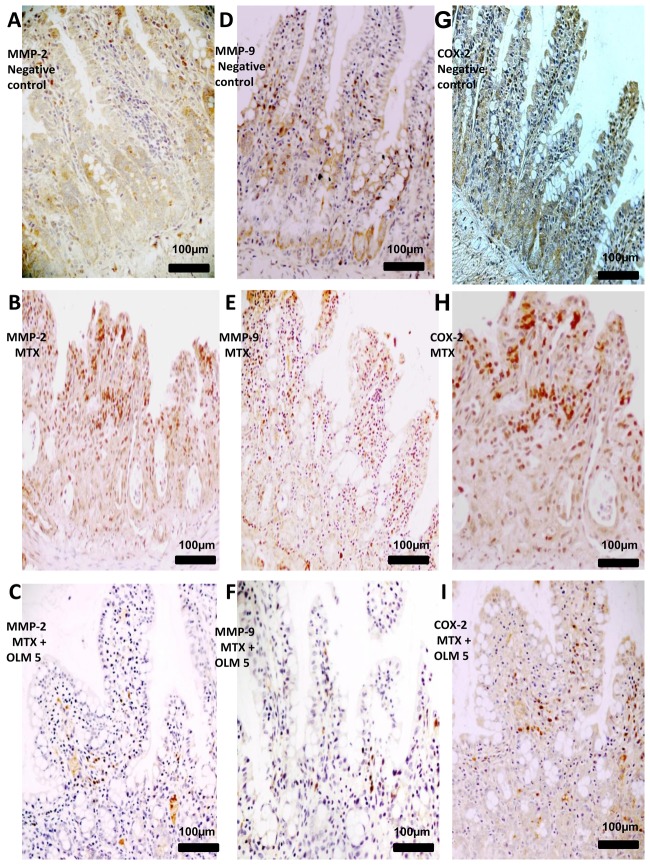
IHC examination of jejunum specimens. For each antigen, three immunolabeled sections were analyzed per animal (N = 5/group). Generally, jejunum from MTX rats had greater MMP-2 (A–C), MMP-9 (D–F) and COX-2 (G–I) immunoreactivity. Magnification 40×, scale bar  = 100 µm.

**Figure 7:**
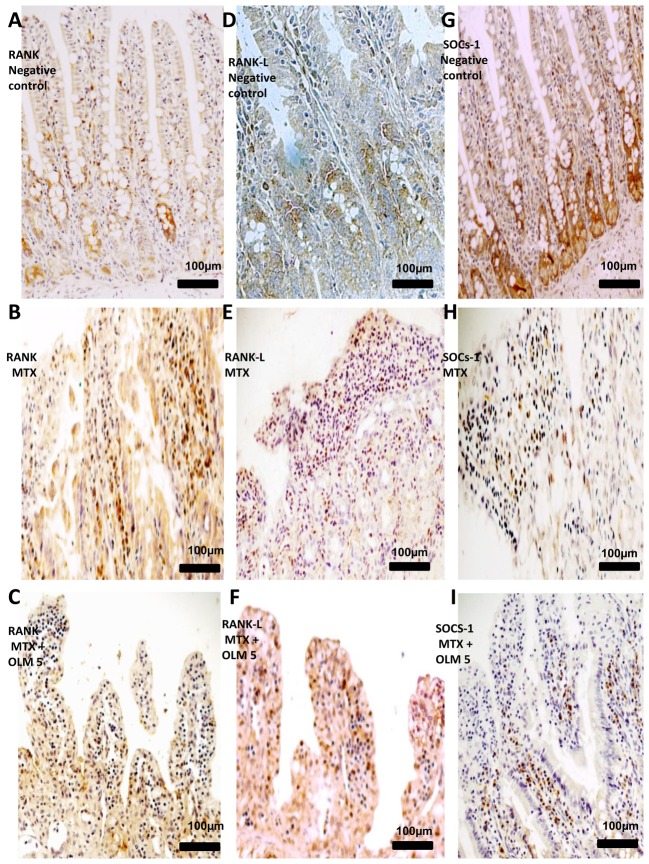
IHC examination of jejunum specimens. For each antigen, three immunolabeled sections were analyzed per animal (N = 5/group). Generally, jejunum from MTX rats had greater RANK (A–C), and RANK-L (D–F) immunoreactivity and reduced SOCs (G–I) immunoreactivity. Magnification 40×, scale bar  = 100 µm.

**Figure 8:**
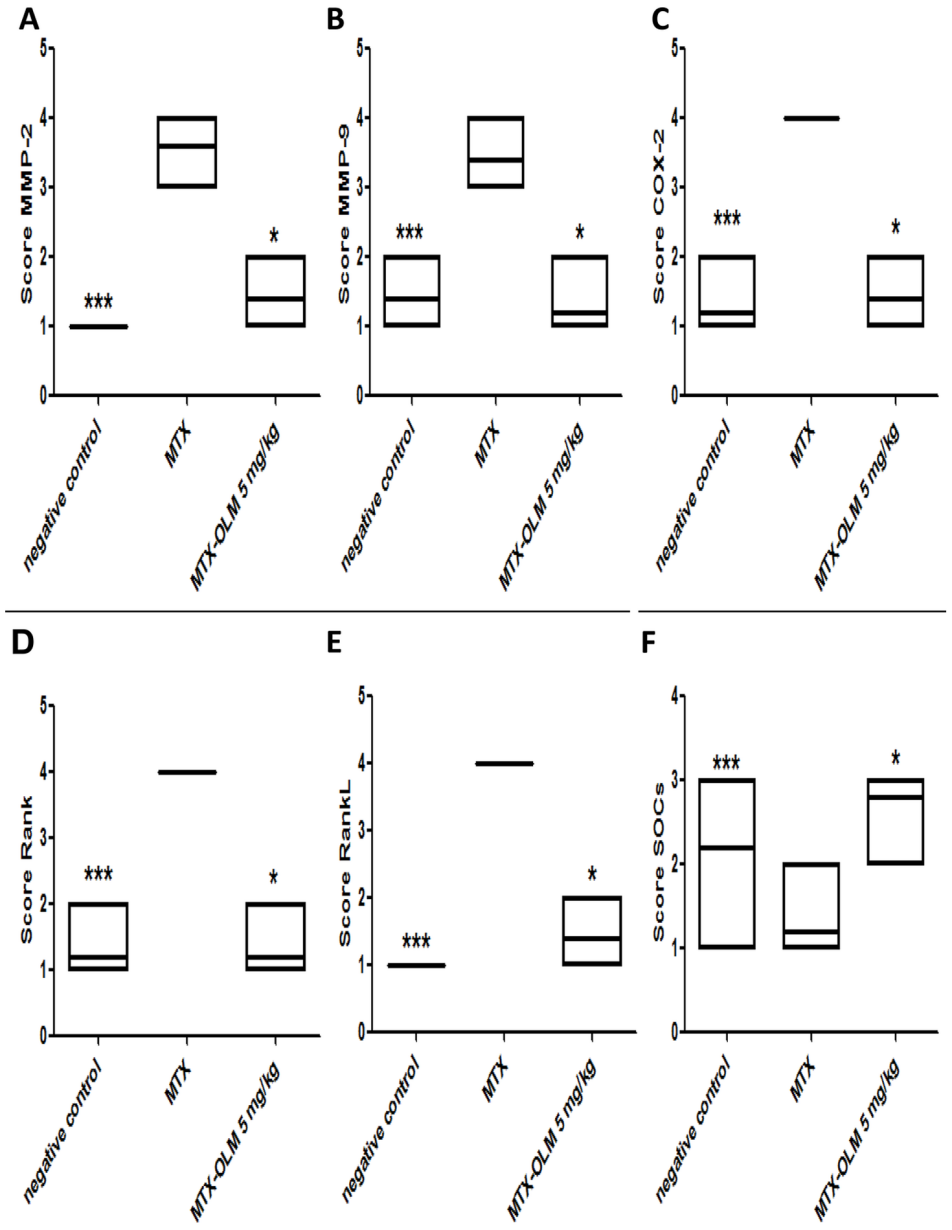
Effect of OLM treatment on intestinal damage in rats. For each group, 5 animals were used, and three immunohistochemistry sections from each animal were analyzed. Representative samples from MTX-OLM 5 mg/Kg treatment group are shown with graphs summarizing the group's mean score, showing immunoreactivity to MMP-2, MMP-9, COX-2, RANK, RANK-L, and SOCs . Note that these effects were reversed (i.e. normalized) in the MTX- OLM 5 mg/kg group's jejunum . *p<.05 vs MTX and ***p<.001 negative control vs. MTX, Kruskal-Wallis test followed by Dunn's test.

We investigated expression levels of MMP-2, MMP-9, COX-2, RANK/RANKL and SOCS-1 in rat intestinal tissues from the negative control, MTX , and MTX-OLM 5 mg/kg groups using immunohistochemistry. The jejunal tissue showed differences in immunohistochemical markers among the groups. Compared to the MTX group, the MTX-OLM 5 mg/kg group exhibited reductions in the levels of MMP-2, MMP-9 (Fig. 6 A,B,C and D,E,F, respectively), COX-2, RANK (Fig. 6 G,H,I and Fig. 7 A,B,C, respectively) and RANKL (Fig. 7 D,E,F) and an increase in the expression the immunosuppressive protein SOCS-1 (Fig. 7 G,H,I). There was a decrease in immunohistochemical scores of MMP-2, MMP-9, COX-2, RANK and RANK-L of group OLM-MTX 5 mg/kg compared to MTX (all, p<0.05 Fig. 8 A,B,C,D and E, respectively ) and an increase in immunohistochemical scores of SOCs of MTX -OLM 5 mg/kg group compared to MTX ( p<0.05 Fig. 8 F).

### Confocal immunofluorescence

**Figure 9:**
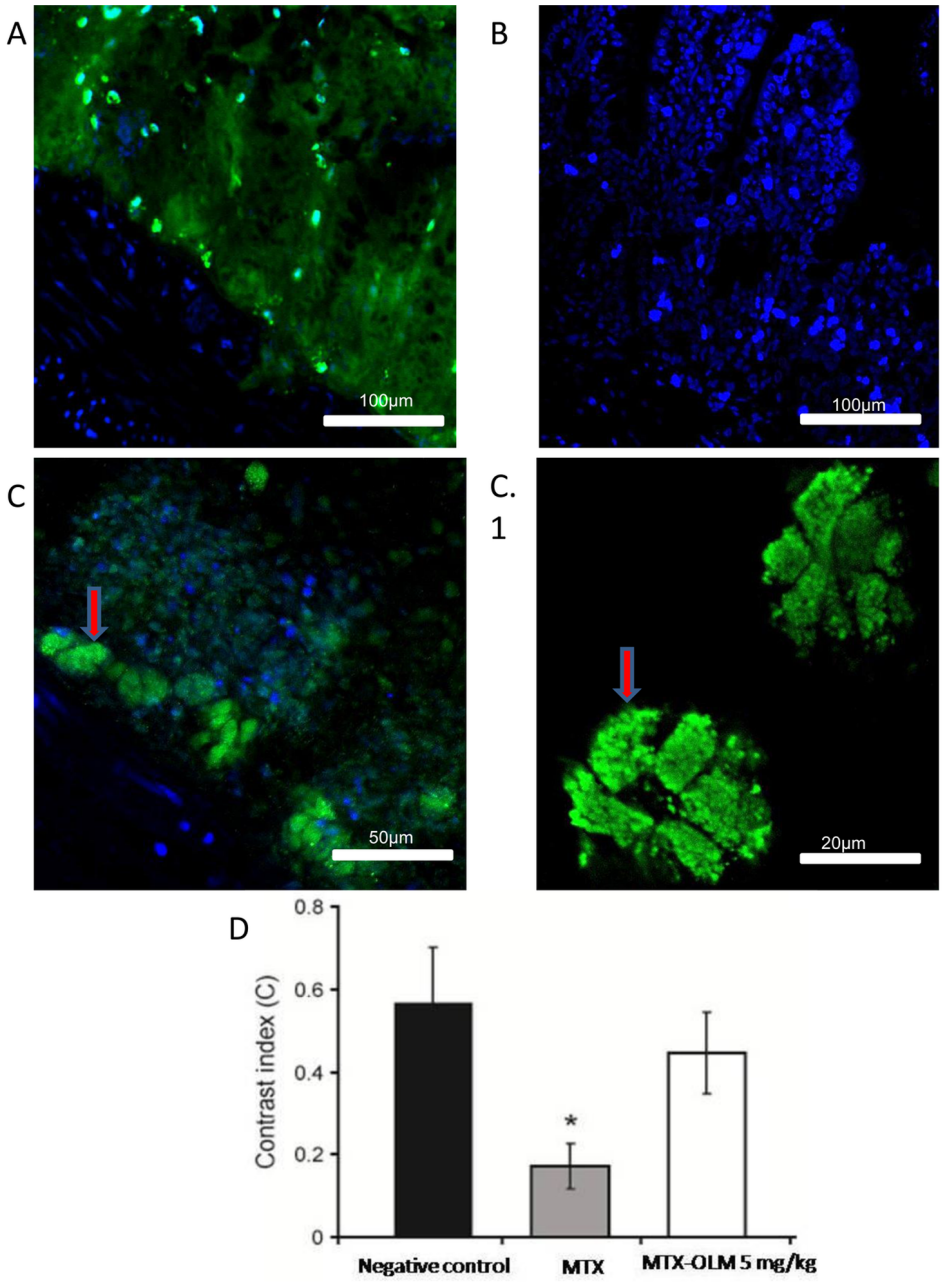
OLM reverses MTX-induced suppression of SOCs-1 protein expression. Representative confocal photomicrographs of SOCs-1 immunoreactivity (green) in jejunum from each group. The sections are nuclear counterstained with DAPI (blue). (A) negative control rat jejunum had moderately diffuse SOCs-1 labeling in all mucosa layers, 20×. (B) SOCs-1 labeling was absent in the MTX intestinal mucositis group, 20×. (C, C.1) Strong cytoplasmic SOCs-1 labeling (red arrows) was seen in the group treated with MTX-OLM 5 mg/kg, 20× and scale bar 50 µm and, 63× and scale bar 20 µm, respectively. (D) Densitometric analysis confirmed a significant reduction in SOCs-1 immunoreactivity in the MTX group that was blocked in the MTX-OLM 5 mg/kg group (*p<.05, Kruskal-Wallis test followed by Dunn's test).

We observed differences between the cellular localisation of the SOCS-1 protein in the negative control, MTX, and MTX-OLM 5 mg/kg groups. The SOCS-1 signal was strongly cytoplasmic (green) in the cells of the group treated with MTX-OLM 5 mg/kg (Fig. 9C and C1), moderately diffuse (green) in all mucosal layers in the negative control group (Fig. 9A), and absent (blue) in the MTX group (Fig. 9B).

## Discussion

Mucositis is an important side effect of MTX therapy. There is no definitive prophylaxis or treatment for mucositis, due in part to a lack of understanding of its pathogenesis and its impact on intestinal structure and function. We used an intestinal mucositis model to analyse the behaviour of the inflammatory process in the presence of an ARB, olmesartan. The anti-inflammatory benefit of the combined use of an immunosuppressive agent (e.g., MTX) with an ARB was previously observed in two experimental models of arthritis [20], [21].

Microscopic signs of intestinal mucositis were observed in the MTX-treated group, including marked detachment of surface epithelium, potentially due to stromal oedema. Additional signs of mucosal damage, including distortion, fusion, shortening and blunting of villi, were observed in rats subjected to MTX treatment, and were associated with a significant decrease in villus height. These changes are consistent with a previous report by Boukhettala, et al [22] . Our histopathological data demonstrated a significant reduction in the presence of these inflammatory markers in the rats treated with MTX- OLM 5 mg/kg. Significant increases in the villus height were observed in the MTX-OLM 5 mg/kg group, compared to the positive control (MTX), suggesting a reduction in intestinal damage in the rats treated with OLM.

We observed an increase in MPO activity in all intestine segments, suggesting participation of neutrophil infiltration in MTX-induced intestinal mucositis, consistent with previous literature. Miyazono et al [23] demonstrated that neutrophil infiltration plays an important role in MTX-induced damage of the small intestine in rats. The inflammatory phase of chemotherapy-induced mucositis is initiated by a disruption in DNA synthesis, which impairs metabolism in the rapidly dividing progenitor cells of the epithelia. This can lead to mitotic inhibition, disruption of cell-cell and cell-substratum interactions, and a slight reduction in epithelial integrity. After these events, pro-inflammatory cytokine levels increase, followed by neutrophil infiltration [24]. We observed a significant reduction in tissue levels of IL-1β and TNF-α, important markers of inflammation following treatment with OLM. Further, previous studies have demonstrated the anti-inflammatory activity of OLM in reducing the levels of these cytokines [12], [25].

Increased levels of pro-inflammatory cytokines, ROS and cyclooxygenase-2 (COX-2) lead to mucosal injury. Increased COX-2 activity in 5-FU- and radiation-induced mucositis suggests an important role for COX-2 in the pathogenesis of oral mucositis [26], [27]. We found that COX-2 activity was increased in the MTX-treated group and decreased in the MTX-OLM 5 mg/kg group.

Matrix metalloproteinases (MMPs) are produced in the GI tract by several structural cells. MMPs are a class of structurally related proteins that are collectively responsible for the metabolism of the extracellular matrix (ECM) of the connective tissue [28]. These zinc- and calcium-dependent endopeptidases degrade most ECM components. They are involved in the remodelling and degradation of matrix components, such as collagen, proteoglycans, and glycoproteins [29].

TNF-α is one of the most important inducers of MMP production. MMPs have been found in inflamed tissues of patients with inflammatory bowel disease, suggesting a role for these enzymes in the increased proteolysis of the mucosa, leading to ulceration, inflammation, and fistula formation [30]. We observed decreased tissue staining for MMP-2 and MMP-9 in the group that was treated with MTX-OLM 5 mg/kg. This finding reflects a reduction in the proteolysis of the mucosa that can lead to ulceration and inflammation of the intestinal tissue. The opposite effect was observed in the MTX group. Increases in MMP-2, -3, -9 and -12 expression have been associated with inflammatory infiltratration and increased tissue damage [31].

Given the role of suppressor of cytokine signaling-1 (SOCS-1) in inhibition of cytokine-mediated signalling, one of the most representative functions of SOCS-1 is to regulate the signalling activated by TLR signalling-induced cytokines [32].

We observed a decrease in the tissue staining of SOCS-1 in the positive control group (MTX), whereas the tissue staining was increased in the group treated with OLM. This finding can be explained by the negative regulatory role of SOCS-1 in the production of pro-inflammatory cytokines. Treatment with MTX-OLM 5 mg/kg stimulates the anti-inflammatory activity of SOCS. Macrophages lacking SOCS-1 have been shown to be hypersensitive to LPS, leading to increased production of pro-inflammatory cytokines [33].

SOCS-1, also called SSI-1 (STAT-induced STAT inhibitor-1) and JAB-1 (JAK binding protein-1) is an intracellular negative-feedback regulator. SOCS-1 inhibits over activation of the JAK-STAT (Janus kinase-signal transducers and activators of transcription) signalling pathway of cytokines by binding to and inhibiting activated JAKs [34]. He et al. showed that SOCS-1 can inhibit TNF-α-induced JNK activation in endothelial cells [35].

Regulatory T (T_R_) cells play a central role in the maintenance of immunological homeostasis. Specific molecular mechanisms control the function of T_R_ cells in the intestine to suppress intestinal inflammation. T_R_ cells preferentially express a TNF family member, receptor activator of NF-κB ligand (RANKL), that blocks the signalling pathway via RANKL and the receptor activator of NF-κB (RANK) [36]. Totsuka et al. showed that the RANK-RANKL signalling pathway is critically involved in regulating the function of T_R_ cells in colitis [37]. Cytoplasmic SOCS-1 has been hypothesised to regulate the transcription factor NF-κB [38]. In our study, we observed an increase in RANK/RANKL expression in the group treated with MTX-OLM 5 mg/kg compared to the MTX group. Presumably, this response is an attempt to regulate the immune response and reduce the inflammatory process.

The group treated with MTX-OLM 5 mg/kg displayed a high level of cytoplasmic expression of SOCS, indicating negative regulatory action on the RANK/RANKL complex that will prevent the nuclear translocation of NF-κB and the production of pro-inflammatory cytokines [39].

One of the disadvantages of chemotherapy is the adverse effect on cells with high mitotic capacity, such as bone marrow cells, and leukopenia is a commonly observed adverse effects of chemotherapy [40], [41]. Our data indicate that the interaction between olmesartan and methotrexate worsened the leukopenia caused by the methotrexate, an effect which may predispose patients to infectious disease. Our findings draw attention to this drug interaction, which has not been reported in the literature, and suggest the importance of careful monitoring of patients on this combination of medications.

Based on these findings, we hypothesise that the beneficial effect of olmesartan treatment is specifically exerted during the damage through blocking inflammatory cytokines in intestinal mucositis.

In summary, MTX inhibits the SOCS-1 signalling pathway, whereas OLM improves intestinal recovery and attenuates the inhibitory effect of MTX on SOCS-1. Specifically, OLM treatment reduces expression of the cytokines IL-1β and TNF-α, down regulates expression of COX-2, MMP-2, MMP-9 and RANKL/RANK, and up regulates expression of SOCS-1. The positive effect of OLM on intestinal structure in intestinal mucositis suggests that OLM treatment may aid in the recovery of oncologic patients receiving chemotherapy, provided the patient is carefully monitored.
