# Correction: Olmesartan Decreased Levels of IL-1β and TNF-α, Down-Regulated MMP-2, MMP-9, COX-2, RANK/RANKL and Up-Regulated SOCs-1 in an Intestinal Mucositis Model

**DOI:** 10.1371/journal.pone.0120057

**Published:** 2015-03-25

**Authors:** The PLOS ONE Staff

The fifth author’s name is spelled incorrectly. The correct name is: Marco Aurélio M. Freire. The correct citation is: Araújo Júnior RFd, da Silva Reinaldo MPO, Brito GAdC, Cavalcanti PdF, Freire MAM, de Medeiros CAX, et al. (2014) Olmesartan Decreased Levels of IL-1β and TNF-α, Down-Regulated MMP-2, MMP-9, COX-2, RANK/RANKL and Up-Regulated SOCs-1 in an Intestinal Mucositis Model. PLoS ONE 9(12): e114923. doi:10.1371/journal.pone.0114923
